# PREDICTING CLIENT RETURN TO ADULT PROTECTIVE SERVICES: A SECONDARY ANALYSIS OF RECURRENCE USING NATIONAL APS DATA

**DOI:** 10.1093/geroni/igad104.0609

**Published:** 2023-12-21

**Authors:** Raphael Gaeta, Zach Gassoumis, Peter Lovegrove

**Affiliations:** New Editions Consulting, Inc., Falls Church, Virginia, United States; University of Southern California, Los Angeles, California, United States; New Editions Consulting, Charlottesville, Virginia, United States

## Abstract

APS programs are provided by state and local governments across the U.S. to serve older adults and adults with disabilities facing abuse, neglect, or exploitation (“maltreatment”). The purpose of this analysis was to identify predictors of recurrence, which occurs when clients return to APS for investigation/services after their cases have been closed. The analysis combined several data sources, including the National Adult Maltreatment Reporting System (NAMRS), the first comprehensive, national data reporting system for APS programs. This analysis represents the first study of this kind to use NAMRS data. The main analyses used Bayesian logistic regression with client, case, and state-level APS system characteristics to predict 12-month recurrence in a multi-state model and in separate single-state models. The sample included data on 1,211,360 APS episodes that closed from 2015-2019 in 19 states. These episodes contained information on 946,477 unique APS clients. About one in five clients in this analysis experienced at least one episode of recurrence. Multivariate results revealed several significant predictors of recurrence (e.g., gender, maltreatment type, maltreatment disposition, episode length, case closure reason), with the largest being age (85+ vs. ≤59 AOR=0.876) and self-neglect (AOR=1.132). The findings may be helpful for APS programs to better recognize and serve clients at high risk of returning to APS after their cases close. The findings also highlight the ambiguity surrounding the concept of recurrence in the APS field and the need for further research to determine the circumstances in which recurrence may be considered a negative or a positive outcome.

